# *Plasmodium vivax* Controlled Human Malaria Infection – Progress and Prospects

**DOI:** 10.1016/j.pt.2016.11.001

**Published:** 2017-02

**Authors:** Ruth O. Payne, Paul M. Griffin, James S. McCarthy, Simon J. Draper

**Affiliations:** 1 The Jenner Institute Laboratories, Old Road Campus Research Building, University of Oxford, Oxford, OX3 7DQ, UK; 2 The Centre for Clinical Vaccinology and Tropical Medicine, University of Oxford, Oxford, OX3 7LE, UK; 3 QIMR Berghofer Medical Research Institute, 300 Herston Rd, Herston, Queensland 4006, Australia; 4 Q-Pharm Pty Ltd, Brisbane, Australia; 5 Department of Medicine and Infectious Diseases, Mater Hospital and Mater Medical Research Institute, Brisbane, Australia; 6 The University of Queensland, Brisbane, Australia

**Keywords:** vivax, CHMI, malaria, IBSM

## Abstract

Modern controlled human malaria infection (CHMI) clinical trials have almost entirely focussed on *Plasmodium falciparum*, providing a highly informative means to investigate host–pathogen interactions as well as assess potential new prophylactic and therapeutic interventions. However, in recent years, there has been renewed interest in *Plasmodium vivax*, with CHMI models developed by groups in Colombia, the USA, and Australia. This review summarizes the published experiences, and examines the advantages and disadvantages of the different models that initiate infection either by mosquito bite or using a blood-stage inoculum. As for *P. falciparum*, CHMI studies with *P. vivax* will provide a platform for early proof-of-concept testing of drugs and vaccines, accelerating the development of novel interventions.

## Trends

Controlled human malaria infection (CHMI) studies provide a valuable means to test the efficacy of antimalarial drugs and vaccines and to study host–pathogen interactions, but have almost exclusively been used for *Plasmodium falciparum*.

CHMI with *P. vivax* has now been successfully conducted in several studies via mosquito bite (sporozoite); however, logistical challenges remain alongside the potential for relapsing infection.

One vaccine efficacy study has now been completed using mosquito-bite CHMI.

More recently, an alternative method of CHMI using an infected blood-stage inoculum has been developed and successfully tested in QIMR Berghofer, Brisbane.

Induced blood-stage malaria (IBSM) will aid testing of blood-stage drugs and vaccines, overcomes some of the logistical challenges associated with mosquito-bite CHMI, and could enable the study of parasite transmission stages.

## Controlled Human Malaria Infection

CHMI with *Plasmodium falciparum* is an established method for evaluating new candidate vaccines and antimalarial drugs in early-phase proof-of-concept clinical trials. The controlled nature of these studies enables trials to be undertaken with small numbers of volunteers with power to investigate efficacy against malaria using a variety of defined end-points, thereby accelerating development of antimalarial drugs 1, 2 and vaccines [3]. CHMI can be initiated by the traditional mosquito-bite method (still frequently used), by the injection of cryopreserved sporozoites, or by an inoculum of blood-stage parasites, so-called induced blood stage malaria (IBSM) 3, 4, 5, 6, 7, 8, 9, 10. *P. falciparum* strains other than the reference clone 3D7 and its parental strain NF54 are now being tested, including the 7G8 laboratory isolate and the Cambodian clone NF135.C10 11, 12. Genetically attenuated parasites that arrest development during the liver stage of infection have now been tested in humans [13]. Most of these studies have been carried out in nonendemic settings, but more recently they have also taken place in endemic countries, in particular through the use of cryopreserved sporozoites 14, 15.

By contrast, modern CHMI with *Plasmodium vivax* has been less utilized, with only a small handful of studies reported in the last few years. In only two of the studies published to date has efficacy of immunization been assessed (Table 1).

There is an extensive history of deliberate infection with *P. vivax* – most notably in malariotherapy, which was carried out for the treatment of neurosyphilis almost a century ago. The Austrian psychiatrist Julius Wagner-Jauregg later received a Nobel Prize for his work with this treatment [16], and the practice was widely adopted as the only effective treatment available at the time. Malariotherapy provided a wealth of information about *P. vivax* infection, which has been reviewed previously [17]. Deliberate infection with *P. vivax* was also conducted in the USA from the 1940s to the 1970s in prisoners involved in the Malaria Research Project at the Illinois State Penitentiary. The studies mainly examined compounds for their potential use as antimalarials [18]. Similar studies were also carried out at the United States Penitentiary, Atlanta, and in both programs the Chesson strain of malaria was used because it was noted to be more likely to relapse and have a shorter latency period than previously utilized strains, meaning that compounds could be assessed more rapidly 18, 19. Key discoveries of the biology of *P. vivax* were made during this period including, for example, the association between Duffy negativity and resistance to *P. vivax* infection [20]. This review focuses on the more recent trials using *P. vivax* CHMI rather than these early studies and treatment programs.

## *P. vivax* Studies Using Sporozoite (Mosquito-Bite) CHMI

Following on from the studies of *P. vivax* infection conducted in Illinois, CHMI experiments were carried out to see if prior exposure to irradiated mosquitoes could confer protection by immunization. Rieckmann *et al.* [21] reported no protection against CHMI in three participants previously exposed to *P. vivax-*infected irradiated mosquitoes on four occasions at intervals of 2–4 weeks (total of <200 mosquitoes).

Three *P. vivax* CHMI studies assessing the ability to ‘immunize’ with X-irradiated sporozoites also took place in Maryland, USA, during the 1970s. Following immunization, challenge infection was initiated by periodic exposure to the bites of nonirradiated infected mosquitoes in three volunteers in separate experiments, and blood films were taken at least daily for all volunteers to assess outcome; between 6 and 14 infected nonirradiated mosquitoes were used for the infection challenges. These experiments in Maryland demonstrated that CHMI with both *P. falciparum* and *P. vivax* could be successfully carried out, but the studies were very small. In these studies, exposure to *P. falciparum* did not confer protection against subsequent CHMI with Chesson strain *P. vivax* 22, 23. However, exposure to X-irradiated Chesson strain and El Savador strain *P. vivax* was able to confer protection against *P. vivax* CHMI following some of the ‘immunization phases’, but not all 22, 23, 24. It was also noted that, unlike the Chesson strain, the newly isolated El Salvador *P. vivax* strain demonstrated long latency. In summary, the pre-patent period (time before development of blood film-detectable parasitemia) for control volunteers in the three experiments ranged between 9 and 16 days. These experiments also demonstrated that protection against *P. vivax* infection could be achieved for a short duration (3–5 months), but required exposure to hundreds of X-irradiated mosquitoes. Recent data emerging from the field of *P. falciparum* sporozoite immunization indicate that the magnitude of protection, longevity of protection, and the ability to protect against heterologous strains requires increasing doses of sporozoites [25]. Future CHMI studies with *P. falciparum* and *P. vivax* may build on these historical experiments to assess whether the same is true for *P. vivax* and even cross-species protection.

More recent mosquito-bite CHMI trials have taken place in Cali, Colombia. The first of these involved 18 healthy volunteers exposed to the bites of two to ten infected *Anopheles albimanus* mosquitoes [26]. *P. vivax* infection was established in mosquito lots fed on blood from 15 patients presenting to outpatient clinics at the Immunology Institute in Cali and Buenaventura with *P. vivax* infection. Four mosquito lots had to be discarded due to coinfection with *P. falciparum*, hepatitis B, or hepatitis C in the donor blood. A mosquito lot that demonstrated a sporozoite rate of 97% the day before CHMI was selected for use in the trial. Seventeen of the 18 volunteers developed *P. vivax* malaria, confirmed by thick blood smear, with a pre-patent period between 9 and 13 days. At diagnosis, volunteers were treated with standard *P. vivax* therapy consisting of chloroquine (600 mg initially, followed by 450 mg 24 and 48 h later) to clear blood-stage infection and primaquine (30 mg/day for 14 days) to achieve radical cure. Levels of parasitemia ranged from 75 to 420 parasites/μL; parasites were cleared within 48 h of starting treatment in all infected volunteers. There were no serious adverse events (SAEs) in this trial, but seven volunteers required fluid therapy due to nausea and vomiting, and five developed blurred vision lasting 2–3 days after treatment initiation. Authors speculated that the volunteer who did not develop malaria had surreptitiously taken antimalarial medication, but this was never confirmed.

The second *P. vivax* CHMI trial was carried out by the same group in Colombia, aiming to demonstrate the reproducibility of this method of infection using three different *A. albimanus* mosquito lots fed on blood from three *P. vivax*-infected donors [27]. Seventeen Duffy-positive individuals and five Duffy-negative controls were enrolled into the study. Participants were randomly assigned to three groups (with six Duffy-positive individuals in two of the groups and five in the third group), and exposed to the bites of two to four infected mosquitoes. The Duffy-negative controls were assigned across the three groups, with two controls in the first two groups and one in the third. All Duffy-positive participants (and none of the Duffy-negative participants) developed blood-stage malaria. The pre-patent period ranged from 9 to 16 days, and was different in the first group, with a median of 14 days, as opposed to a median of 10 days in the second two groups.

A third *P. vivax* CHMI trial was carried out in Cali but this time among both ‘semi-immune’ (previously-exposed; n = 9) and malaria-naïve adult volunteers (n = 7) [28]. Previous exposure was confirmed by clinical history and a positive indirect fluorescent antibody test against *P. vivax* blood stages. A mosquito lot from one of six *P. vivax*-infected patients was used for all volunteers, who were each exposed to bites from two to four infected mosquitoes. The pre-patent periods ranged between 11 and 13 days, with no significant difference between the malaria-naïve and ‘semi-immune’ volunteers. Symptoms were significantly worse among the malaria-naïve subjects but there were no SAEs. One malaria-naïve volunteer developed parasitemia detectable by real-time quantitative polymerase chain reaction (RT-qPCR) by day 9, but cleared the parasitemia spontaneously in 4 days. An antimalarial drug screen was negative; the authors speculated that this phenomenon may be due to Duffy antigen polymorphism. Another malaria-naïve volunteer presented 3 months after treatment with a *P. vivax* infection after visiting an endemic area. However, the investigators were unable to determine whether this episode of malaria was due to reinfection or relapse, as the volunteer had visited the same endemic area that the CHMI strain had come from.

A subsequent analysis of differences in gene expression between malaria-naïve and ‘semi-immune’ volunteers demonstrated significant changes in gene expression at the time of malaria diagnosis, particularly in the naïve volunteers, with downregulation of multiple genes related to innate immunity, inflammation, and neutrophil abundance [29]. Antibody profiling was also undertaken, using a custom protein microarray. This demonstrated increased responses in the semi-immune participants compared with naïve individuals before CHMI, although responses in both groups were higher than those from US controls. Following CHMI, antibody responses increased on day 45 and had declined to near baseline by day 145. Volunteers who experienced fever were found to have significantly higher responses to the *P. vivax* antigens merozoite surface protein 3 (MSP3), MSP4, MSP5, and MSP10 at the day 45 time-point [30].

The most recent CHMI trial from the Cali group [31] involved the use of radiation-attenuated sporozoites in Duffy-positive and Duffy-negative healthy adult volunteers, delivered by mosquito bite. Mosquitoes were infected as described above for the other trials conducted by this group. Sporozoites were attenuated by exposing the mosquitoes to 150 ± 10 cGy of gamma irradiation. Twelve Duffy-positive participants and five Duffy-negative controls completed the immunization phase with exposure to *P. vivax*-infected irradiated *A. albimanus* mosquitoes followed by CHMI. Two Duffy-positive controls also completed the trial; these participants were exposed to nonirradiated, noninfected mosquitoes. Seven immunizations were carried out for each volunteer on weeks 0, 8, 12, 23, 48, 51, and 56, with a mean of 65 infectious bites for each immunization. Two weeks after the final immunization, participants were treated with chloroquine and primaquine to clear any malaria infections that may have developed during the immunization phase. Plasma levels of chloroquine and primaquine were checked prior to CHMI to ensure drug clearance. CHMI was carried out at week 64 using two to four *P. vivax*-infected mosquito bites, and participants were monitored daily with thick blood films from day 6. There were no reported SAEs related to immunization, although one volunteer developed severe elevation of hepatic transaminases [>10 times the upper limit of normal (x ULN)] with associated abdominal pain and vomiting following CHMI, with no alternative cause found. These symptoms resolved spontaneously. The protective efficacy of the immunization schedule was 42% (five out of twelve Duffy-positive participants protected). In volunteers who developed malaria, the mean pre-patent period until thick blood film positivity was 12.8 days. Interestingly, all of the volunteers protected in this trial were female [31].

Only one other CHMI trial assessing a *P. vivax* vaccine has been published to date. The VMP001/AS01B vaccine was tested in healthy malaria-naïve adults at the Walter Reed Army Institute of Research (WRAIR) in the USA [32]. VMP001 is a soluble recombinant protein vaccine [33], encoding the *P. vivax* circumsporozoite protein (PvCSP), administered with the AS01B adjuvant (GlaxoSmithKline). The vaccine was administered to 30 volunteers in three cohorts (10 in each) at doses of 15 μg, 30 μg, and 60 μg, given three times at a 4-week interval between the first and second dose; the third dose was given 8 (15 μg cohort), 6 (30 μg cohort), or 4 (60 μg cohort) weeks after the second. Twenty-nine volunteers completed the vaccination phase, with 27 proceeding to CHMI 2 weeks after final vaccination, along with six malaria-naïve controls. Mosquito-bite CHMI with five *P. vivax*-infected *Anopheles dirus* mosquitoes was undertaken. Laboratory-reared mosquitoes were fed on blood from a *P. vivax*-infected donor in Thailand after screening by PCR to ensure no coinfection with other *Plasmodium* species or blood-borne infections. Infected mosquitoes were then transported to WRAIR and maintained in their insectary until CHMI. Volunteers were treated following a diagnosis of vivax malaria by thick blood smear. The vaccine protective efficacy was 0%; all volunteers had developed thick blood film-detectable parasitemia by day 13. The median pre-patent period for all immunized participants was 11.9 days versus 10.7 days for infectivity controls. Participants were treated with standard chloroquine and primaquine therapy with rapid clearance of infection. However, two volunteers went on to have multiple relapses. One participant experienced two relapses (at weeks 8 and 18 after CHMI), while the other experienced three (at weeks 11, 20, and 48 after CHMI) [34]. By study completion, the participants had been followed up for 5 years, and had not had any further relapses [32]. Exploratory genotyping for the cytochrome P450 (CYP) allele CYP2D6 was undertaken in 25 of the 33 volunteers. The volunteers with relapses were found to have either an intermediate-metabolizer phenotype or poor-metabolizer phenotype. These phenotypes were associated with significantly lower levels of primaquine clearance 24 h after dosing [34]. Primaquine is metabolized into redox-active metabolites by CYP2D6, and therefore individuals who are unable to metabolize the drug in sufficient quantities appear to be at risk of relapse from *P. vivax*.

Another CSP *P. vivax* vaccine (CS long synthetic peptides formulated in Montanide ISA 51) is currently being assessed in a mosquito-bite CHMI trial (NCT02083068), but no results have yet been published.

## Studies Using Blood-Stage CHMI

There have been four *P. vivax* IBSM/blood-stage CHMI studies to date successfully carried out at QIMR Berghofer in Brisbane, Australia, two of which have been published. The first trial was a proof-of-concept study with only two volunteers, using an inoculum collected from a donor whose blood group was A, Rh negative, Duffy-positive who had travelled to the Solomon Islands and returned with clinical vivax malaria, designated HMPBS01-Pv [35]. This demonstrated that infection with *P. vivax* malaria could successfully be achieved from a frozen inoculum, as has been demonstrated with *P. falciparum* for many years 5, 6. The two volunteers were injected intravenously with around 13 000 genome equivalents of *P. vivax* and developed parasitemia detectable by qPCR on days 8–9, with a peak parasitemia on the day of treatment (day 14) in both individuals. In this case, the challenge inoculum cannot be cultured by limiting dilution to determine the parasite dose (as is routinely done for *P. falciparum*) 7, 36, and hence a qPCR-based method was used to quantify the inoculum. Both subjects developed symptoms consistent with early malaria infection. Volunteers were treated with a standard treatment course of artemether–lumefantrine (four tablets each containing 20 mg artemether and 120 mg lumefantrine every 12 h for six doses), with a subsequent rapid decline in parasitemia. Of note, the presence of a marker of mature gametocytes was detectable in the blood of both volunteers by qPCR on days 11–12, prior to the onset of symptoms in one volunteer. In this case the gametocyte marker was *pvs25* transcripts – these are expressed by the parasite shortly before gametocyte fertilization with synthesis peaking over the proceeding hours.

The second study utilized the same inoculum in a further six subjects and was designed to validate the pilot study model and evaluate the potential for transmission to vector mosquitoes [37]. The mean dose of parasites (± SD) administered to the six subjects in this study was 31 786 (± 11 947) parasites as determined by qPCR testing of the inoculum, whilst linear regression modelling of the *in vivo* qPCR parasite growth data estimated the starting dose of viable parasites to be a mean ± SEM of 15 ± 5 parasites. The kinetics of the parasitemia (determined by qPCR) were similar in all volunteers with first detection on day 8 in four subjects and day 9 in two subjects. Peak parasitemia occurred on day 14 (the day of antimalarial treatment) with a median of 31 parasites/μL. The parasite multiplication rate (PMR), assuming a 48-hour lifecycle, was 9.9 (95% CI: 7.7–12.4). The clinical course was also similar in all volunteers, with the mean onset of symptoms occurring on day 12.2 (range 11–13).

There were no SAEs reported in this study; most of the adverse events were mild expected symptoms of malaria. However, four of the six subjects demonstrated significant elevations of hepatocellular enzymes (>5 x ULN), although asymptomatic and not associated with significant elevations in bilirubin (Hy's law was not met) (http://www.fda.gov/downloads/Drugs/GuidanceComplianceRegulatoryInformation/Guidances/UCM174090.pdf). The specific cause of the elevated hepatocellular enzymes was not conclusively determined, but extensive additional investigations, including viral serologies (hepatitis viruses, herpes viruses, alphaviruses and flaviviruses), other biomarkers (creatinine kinase, paracetamol levels), and liver ultrasound did not demonstrate any significant abnormalities. The inoculum (and donor) had been screened to exclude the presence of blood-borne pathogens other than malaria to a greater level than required by the local blood service. All subjects were treated with artemether–lumefantrine on study day 14 with a corresponding rapid decline in parasitemia and clinical symptoms.

There was no evidence of reactions to the donor's blood, that is, no transfusion reactions and no red cell alloantibodies detected, and no evidence of transmitted infection (other than malaria). Whilst the initial *P. vivax* isolate demonstrated a favourable profile in two studies, it was obtained from a blood group A donor. Therefore, to reduce the probability of alloantibody generation and to optimize the probability of infection, it is required that recipients are also blood group A (and Duffy positive). This significantly limits the number of eligible volunteers. To overcome this limitation, and as a result of an ongoing cryobanking project, a second *P. vivax* isolate was obtained from a blood group O patient who returned to Brisbane, Australia, having acquired *P. vivax* infection in India. This inoculum has since been used in two further studies (ACTRN12614000930684 and ACTRN12616000174482), but results have not yet been published.

The development of gametocytemia in volunteers undergoing CHMI has implications for carrying out such studies in areas of the world where *Anopheles* mosquito vectors are present and capable of supporting transmission. Of note, *A. albimanus* mosquitoes fed on blood from infected volunteers between day 7 post-CHMI and the day of diagnosis (days 11–13) in the third trial carried out in Cali, Columbia [28], did not develop oocysts over the next 7 days. This was despite the presence of parasites, likely to be gametocytes, being identified in the circulation as early as day 7 by qPCR for two sexual stage-specific molecular markers, *pvs25* and *pvs16*. Direct (skin) feeding was carried out as well as via a membrane feeding assay. In contrast, parasites obtained from submicroscopic asymptomatic and naturally infected individuals were able to infect mosquitoes [38]. This difference is thought to be due to insufficient maturation of gametocytes at the point of diagnosis in the early CHMI infections.

The second blood-stage CHMI trial in Brisbane showed more promise for assessment of transmission [37]. In this trial, detection of *pvs25* transcripts was used as a marker of gametocytemia. Expected kinetics relative to the parasitemia were observed, with peak *pvs25* detection occurring immediately prior to antimalarial treatment (day 14), with a median of 4.90 × 10^5^ transcripts/mL (range from 3.96 × 10^4^ to 2.37 × 10^6^). The potential for transmission to mosquito vectors was assessed by direct (skin) and membrane mosquito feeding for 2 to 3 days prior to antimalarial therapy. A total of 16 direct feeding assays and 32 membrane feeding assays were conducted. A total of 1801 *Anopheles stephensi* mosquitoes were dissected for the detection of oocysts, and a low prevalence of mosquito infection was observed (1.8%; n = 32/1801 mosquitos). Moreover, unlike *P. falciparum, P. vivax* gametocytes are susceptible to asexual-stage antimalarial treatment so any gametocytes should also be cleared following initiation of standard therapy [39].

In line with these experiences, a recently developed simulation model (using data from a previously developed model of red blood cell invasion and information from six published time series of parasitemia from neurosyphilis patients treated with *P. vivax*) further supports these findings. Data suggest that a density of 116 gametocytes/μL is required for there to be at least one male and one female gametocyte in a 1 μL blood meal, and this model predicts that this threshold will first be reached in an infected naïve host at 7.9 days post patency on average (95% CI 5–10) [40]. Overall, these data suggest that transmission efficiency during standard CHMI protocols would likely be very low.

## Comparison of CHMI Models

Modern CHMI studies have proved extremely useful in early-phase testing of *P. falciparum* vaccines 3, 6, 41, 42, 43, 44, 45, 46 and drugs 1, 2, 47, 48. The development of modern CHMI approaches for *P. vivax* is less well established, but has the potential to provide a useful and cost-effective means of early-phase analysis of antimalarial drugs and vaccines. To date, the only methods trialled in humans are CHMI by mosquito bite or by intravenous inoculation of parasitized red blood cells. Cryopreserved sporozoites, as developed by Sanaria, are another potential method for CHMI, but unlike *P. falciparum* cryopreserved sporozoites (PfSPZ Challenge), have not yet been used in any human trials (http://projectreporter.nih.gov/project_info_description.cfm?aid=8446961).

There are advantages and limitations to both available CHMI models (see Outstanding Questions). Mosquito-bite CHMI most closely resembles natural infection but requires fresh gametocytes from an infected patient due to the unavailability of long-term *P. vivax* culture. This requires at least part of the trial to be undertaken in an endemic setting to screen and enrol patients, with appropriate entomological facilities established to produce an infected mosquito lot. Subsequently, the mosquito lot can be used in trials in the same location, or transported to nonendemic areas. Ensuring the successful production of infected mosquitoes, in conjunction with recruitment of volunteers who may receive an intervention such as a vaccine, poses significant logistical challenges, especially if the timing of the vaccination and subsequent CHMI are critical. Moreover, a different isolate of *P. vivax* will inevitably be used for every trial, meaning that CHMI assessment of vaccines is almost certainly to be with a heterologous strain to that used in the vaccine; that the parasites may have different susceptibility to antimalarial treatment between strains; and these differences will be unknown at the time of CHMI. As seen in the trials carried out in Cali, different strains are likely to have different pre-patent periods which can limit comparability between trials [27]. The use of sporozoites for CHMI also necessitates a liver stage of infection, with a high risk of hypnozoite formation and potential relapse. This requires participants to be screened for glucose-6-phosphate dehydrogenase (G6PD) deficiency in order to avoid hemolysis induced by primaquine, and now also requires assessment of the volunteers’ ability to metabolize primaquine to maximize safety. Volunteers with poor or intermediate metabolizer CYP2D6 phenotypes should not be enrolled for such studies in the future.

The use of IBSM/blood-stage CHMI does not mimic natural infection but has several advantages over mosquito-bite CHMI. Practical advantages include the ability to carry out CHMI studies more easily in a nonendemic setting; having access to the *P. vivax* strain genetic data before CHMI; being able to carry out multiple studies with the same strain (for which a safety database can be established); and being able to use the same inoculum size for each volunteer or even vary the dose of parasites administered if required. There are also advantages for participants with this method – the use of blood-stage parasites means that there is no liver stage of infection, and therefore no risk of hypnozoite formation or relapse. This means that participants do not require primaquine treatment, and therefore do not require G6PD deficiency or CYP2D6 phenotype screening. Although the numbers of participants who have undergone this method of CHMI are still currently very small, all have successfully been infected with an expected rise in parasitemia detected by qPCR multiplication, unlike in two of the five reported mosquito-bite CHMI studies. The ability to give a small known inoculum directly into the bloodstream also means that the blood stage of infection can be observed for longer (than following mosquito-bite CHMI), and differences in PMR are more likely to be observed between participants and controls. A recent blood-stage *P. falciparum* CHMI trial in Oxford has demonstrated greater power to see a reduction in PMR with smaller numbers of volunteers as compared with sporozoite CHMI studies [36]. Blood-stage CHMI is thus particularly advantageous for the assessment of antimalarial drugs and vaccines which target the erythrocytic stage of the malaria parasite. The model is of course unable to be used for assessment of pre-erythrocytic interventions which does limit its utility, and whether either model can be optimized to robustly assess transmission-blocking interventions remains to be determined.

## Concluding Remarks and Future Perspectives

The development of CHMI models for *P. vivax* is a vital step towards early-phase vaccine and drug testing, allowing relatively small and inexpensive Phase II efficacy trials to take place before larger field trials. The use of *P. vivax* CHMI is more complex than the now established *P. falciparum* models because of inherent differences between the parasites’ biology and, in particular, the inability to maintain *P. vivax* in long-term culture. Mosquito-bite CHMI currently requires access to infected patients and entomology facilities, and leads to the use of genetically variable isolates in each study. The use of cryopreserved sporozoites in the future may represent a particularly useful advancement, but this approach has yet to reach clinical testing. Other approaches may include the use of *P. falciparum* parasites transgenic for antigens of interest to allow for easier CHMI assessment of sporozoite or liver-stage vaccine candidates. To date only one clinical study has been reported using a genetically modified *P. falciparum* parasite, in this case a knockout parasite line [13]. Transgenic parasites have been developed for use in preclinical and *in vitro* studies against *P. vivax*, so there is potential for development for clinical use 49, 50.

Blood-stage *P. vivax* CHMI remains in its infancy, but will likely be more widely used for novel vaccine and drug testing in the coming years. To date, only one vaccine targeting the *P. vivax* blood-stage merozoite – using the chimpanzee adenovirus serotype 63 (ChAd63) and modified vaccinia virus Ankara (MVA) viral vectors encoding the *P. vivax* Duffy-binding protein region II (PvDBP_RII) in a heterologous prime-boost regimen [51] – has completed Phase Ia clinical testing in the UK (NCT01816113) and could progress to Phase IIa efficacy testing. A second protein-based PvDBP_RII vaccine formulated in SE-GLA adjuvant has recently entered Phase I clinical trial in India (CTRI/2016/09/007289). The demonstration of a blood-stage CHMI model for vaccine testing, using *P. falciparum* [36], should allow for similar advances with *P. vivax* in the future. This model may also allow for studies of naturally acquired *P. vivax* immunity if trial centres are established in endemic areas, as is starting to occur now for *P. falciparum* in Africa 14, 15. The development of multiple banks of cryopreserved strains will also allow for vaccine testing against homologous and heterologous strains potentially originating from different geographic areas. CHMI trials can also be used to further investigate the mechanism, immunity, and host–pathogen interactions under controlled infection conditions in humans – the ultimate animal model. These endeavours should help to provide essential new tools to aid in the fight against *P. vivax*, as efforts continue to move towards local malaria elimination.

Outstanding Questions Can *P. vivax* CHMI models be developed to allow for robust testing of blood-stage vaccines and transmission-blocking interventions?

Can a bank of different blood-stage *P. vivax* isolates be established to allow for routine CHMI/IBSM studies using defined, but genetically heterogeneous, parasites?

Can cryopreserved *P. vivax* sporozoites be established to initiate CHMI, potentially using an isolate that minimizes risk of relapsing infection?

What efficacy outcome measures following CHMI would be sufficient, for different types of intervention, to warrant further clinical development and/or testing in the field?

Can *P. vivax* CHMI in naturally immune or endemic populations provide greater insight into mechanisms of immunity to this parasite?

Can screening for volunteers’ cytochrome P450 isoenzyme (CYP2D6) genotype/phenotype identify those more likely to fail primaquine therapy and prevent relapse following *P. vivax* sporozoite CHMI?

## Figures and Tables

**Table 1:** Overview of Published *Plasmodium vivax* CHMI Studies

Trial site	Number of volunteers	Pre-patent period (days)^a^	Number of infected mosquitoes OR infective inoculum	Number of volunteers with patent parasitemia	Refs
Sporozoite (mosquito-bite) CHMI studies					
Cali, Columbia	18	9–13	2–10	17/18^b^	[26]
Cali, Columbia	17 Duffy positive 5 Duffy negative	9–16	2–4	17/17 (Duffy positive) 0/5 (Duffy negative)	[27]
Cali, Columbia	7 malaria-naïve 9 semi-immune	11–13	2–4	16/16^c^	[28]
Cali, Columbia	12 Duffy -positive vaccinees 2 Duffy-positive controls 5 Duffy-negative controls	12–13	2–4	7/12 vaccinees 2/2 Duffy-positive controls 0/5 Duffy-negative controls	[31]
WRAIR, USA	27 vaccinees 6 infectivity controls	10–13 10–11	5	27/27 vaccinees 6/6 controls	[32]
Blood-stage CHMI studies (IBSM)					
QIMRB, Australia	2	8–9	13 000 genome equivalents	2/2	[35]
QIMRB, Australia	6	8–9	31 786 (± 11 947) as determined by qPCR (= 15 ± 5 viable *P. vivax* parasites)	6/6	[37]

a The pre-patent period refers to the period before malaria diagnosis which was made by blood film in sporozoite (mosquito-bite) studies and qPCR in the blood-stage studies.

b One volunteer did not develop parasitemia; the authors of the study suggested that this may have been due to surreptitious self-administration of antimalarial medication, but this was not proven.

c One volunteer developed parasitemia detectable by qPCR but cleared it spontaneously within 4 days.

## References

[1] McCarthy J.S. (2011). A pilot randomised trial of induced blood-stage *Plasmodium falciparum* infections in healthy volunteers for testing efficacy of new antimalarial drugs. PLoS One.

[2] Pasay C.J. (2016). Piperaquine monotherapy of drug-susceptible *Plasmodium falciparum* infection results in rapid clearance of parasitemia but is followed by the appearance of gametocytemia. J. Infect. Dis..

[3] Sauerwein R.W. (2011). Experimental human challenge infections can accelerate clinical malaria vaccine development. Nat. Rev. Immunol..

[4] Sheehy S.H. (2013). optimising controlled human malaria infection studies using cryopreserved parasites administered by needle and syringe. PLoS One.

[5] Engwerda C.R. (2012). Experimentally induced blood stage malaria infection as a tool for clinical research. Trends Parasitol..

[6] Duncan C.J., Draper S.J. (2012). Controlled human blood stage malaria infection: current status and potential applications. Am. J. Trop. Med. Hyg..

[7] Duncan C.J. (2011). Impact on malaria parasite multiplication rates in infected volunteers of the protein-in-adjuvant vaccine AMA1-C1/Alhydrogel + CPG 7909. PLoS One.

[8] Cheng Q. (1997). Measurement of *Plasmodium falciparum* growth rates in vivo: a test of malaria vaccines. Am. J. Trop. Med. Hyg..

[9] Roestenberg M. (2013). Controlled human malaria infections by intradermal injection of cryopreserved *Plasmodium falciparum* sporozoites. Am. J. Trop. Med. Hyg..

[10] Mordmuller B. (2015). Direct venous inoculation of *Plasmodium falciparum* sporozoites for controlled human malaria infection: a dose-finding trial in two centres. Malaria J..

[11] Stanisic D.I. (2015). Development of cultured *Plasmodium falciparum* blood-stage malaria cell banks for early phase in vivo clinical trial assessment of anti-malaria drugs and vaccines. Malaria J..

[12] Teirlinck A.C. (2013). NF135.C10: a new *Plasmodium falciparum* clone for controlled human malaria infections. J. Infect. Dis..

[13] Spring M. (2013). First-in-human evaluation of genetically attenuated *Plasmodium falciparum* sporozoites administered by bite of *Anopheles* mosquitoes to adult volunteers. Vaccine.

[14] Hodgson S.H. (2014). Evaluating controlled human malaria infection in Kenyan adults with varying degrees of prior exposure to *Plasmodium falciparum* using sporozoites administered by intramuscular injection. Front. Microbiol..

[15] Shekalaghe S. (2014). Controlled human malaria infection of Tanzanians by intradermal injection of aseptic, purified, cryopreserved *Plasmodium falciparum* sporozoites. Am. J. Trop. Med. Hyg..

[16] Whitrow M. (1990). Wagner-Jauregg and fever therapy. Med. History.

[17] Snounou G., Perignon J.L. (2013). Malariotherapy – insanity at the service of malariology. Adv. Parasitol..

[18] Alving A.S. (1948). Procedures used at Stateville penitentiary for the testing of potential antimalarial agents. J. Clin. Invest..

[19] Coatney H.R. (1949). Studies in human malaria; trials of quinacrine, colchicine (SN 12,080) and quinine against Chesson strain vivax malaria. Am. J. Hyg..

[20] Miller L.H. (1976). The resistance factor to *Plasmodium vivax* in blacks. The Duffy-blood-group genotype, FyFy. N. Engl. J. Med..

[21] Rieckmann K.H. (1979). Current considerations in vaccination of humans against malaria: Use of attenuated sporozoites in the immunization of human volunteers against falciparum malaria. Bull. Wld. Hlth. Organ..

[22] Clyde D.F. (1975). Immunization of man against falciparum and vivax malaria by use of attenuated sporozoites. Am. J. Trop. Med. Hyg..

[23] Clyde D.F. (1973). Specificity of protection of man immunized against sporozoite-induced falciparum malaria. Am. J. Med. Sci..

[24] McCarthy V.C., Clyde D.F. (1977). *Plasmodium vivax*: correlation of circumsporozoite precipitation (CSP) reaction with sporozoite-induced protective immunity in man. Exp. Parasitol..

[25] Hoffman S.L. (2015). The march toward malaria vaccines. Am. J. Prev. Med..

[26] Herrera S. (2009). Successful sporozoite challenge model in human volunteers with *Plasmodium vivax* strain derived from human donors. Am. J. Trop. Med. Hyg..

[27] Herrera S. (2011). Consistent safety and infectivity in sporozoite challenge model of *Plasmodium vivax* in malaria-naive human volunteers. Am. J. Trop. Med. Hyg..

[28] Arevalo-Herrera M. (2014). *Plasmodium vivax* sporozoite challenge in malaria-naive and semi-immune Colombian volunteers. PLoS One.

[29] Rojas-Pena M.L. (2015). Transcription profiling of malaria-naive and semi-immune Colombian volunteers in a *Plasmodium vivax* sporozoite challenge. PLoS Negl. Trop. Dis..

[30] Arevalo-Herrera M. (2016). Antibody Profiling in Naive and Semi-immune Individuals Experimentally Challenged with *Plasmodium vivax* Sporozoites. PLoS Negl. Trop. Dis..

[31] Arevalo-Herrera M. (2016). Protective efficacy of *Plasmodium vivax* radiation-attenuated sporozoites in Colombian volunteers: a randomized controlled trial. PLoS Negl. Trop. Dis..

[32] Bennett J.W. (2016). Phase 1/2a Trial of *Plasmodium vivax* malaria vaccine candidate VMP001/AS01B in malaria-naive adults: safety, immunogenicity, and efficacy. PLoS Negl. Trop. Dis..

[33] Vanloubbeeck Y. (2013). Comparison of the immune responses induced by soluble and particulate *Plasmodium vivax* circumsporozoite vaccine candidates formulated in AS01 in rhesus macaques. Vaccine.

[34] Bennett J.W. (2013). Primaquine failure and cytochrome P-450 2D6 in *Plasmodium vivax* malaria. N. Engl. J. Med..

[35] McCarthy J.S. (2013). Experimentally induced blood-stage *Plasmodium vivax* infection in healthy volunteers. J. Infect. Dis..

[36] Payne R.O. (2016). Demonstration of the blood-stage *Plasmodium falciparum* controlled human malaria infection model to assess efficacy of the *P. falciparum* apical membrane antigen 1 vaccine, FMP2.1/AS01. J. Infect. Dis..

[37] Griffin P. (2016). Safety and reproducibility of a clinical trial system using induced blood stage *Plasmodium vivax* infection and its potential as a model to evaluate malaria transmission. PLoS Negl. Trop. Dis..

[38] Vallejo A.F. (2016). *Plasmodium vivax* gametocyte infectivity in sub-microscopic infections. Malaria J..

[39] Pukrittayakamee S. (2008). Effects of different antimalarial drugs on gametocyte carriage in *P. vivax* malaria. Am. J. Trop. Med. Hyg..

[40] Kerlin D.H., Gatton M.L. (2015). A simulation model of the within-host dynamics of *Plasmodium vivax* infection. Malaria J..

[41] Roestenberg M. (2012). Efficacy of preerythrocytic and blood-stage malaria vaccines can be assessed in small sporozoite challenge trials in human volunteers. J. Infect. Dis..

[42] Spring M. (2014). Controlled human malaria infection. J. Infect. Dis..

[43] Chulay J.D. (1986). Malaria transmitted to humans by mosquitoes infected from cultured *Plasmodium falciparum*. Am. J. Trop. Med. Hyg..

[44] Ballou W.R. (1987). Safety and efficacy of a recombinant DNA Plasmodium falciparum sporozoite vaccine. Lancet.

[45] Herrington D.A. (1987). Safety and immunogenicity in man of a synthetic peptide malaria vaccine against *Plasmodium falciparum* sporozoites. Nature.

[46] Church L.W. (1997). Clinical manifestations of *Plasmodium falciparum* malaria experimentally induced by mosquito challenge. J. Infect. Dis..

[47] Berman J.D. (2001). Causal prophylactic efficacy of atovaquone-proguanil (Malarone) in a human challenge model. Trans. R. Soc. Trop. Med. Hyg..

[48] Shapiro T.A. (1999). Prophylactic activity of atovaquone against *Plasmodium falciparum* in humans. Am. J. Trop. Med. Hyg..

[49] Somsak V. (2011). Transgenic *Plasmodium* parasites stably expressing *Plasmodium vivax* dihydrofolate reductase-thymidylate synthase as in vitro and in vivo models for antifolate screening. Malaria J..

[50] Mizutani M. (2014). Baculovirus-vectored multistage Plasmodium vivax vaccine induces both protective and transmission-blocking immunities against transgenic rodent malaria parasites. Infect. Immun..

[51] de Cassan S.C. (2015). Preclinical assessment of viral vectored and protein vaccines targeting the Duffy-binding protein region II of *Plasmodium Vivax*. Front. Immunol..

